# Optimization of Targeted Plant Proteomics Using Liquid Chromatography with Tandem Mass Spectrometry (LC-MS/MS)

**DOI:** 10.1021/acsagscitech.3c00017

**Published:** 2023-04-17

**Authors:** Weiwei Li, Arturo A. Keller

**Affiliations:** Bren School of Environmental Science and Management, University of California at Santa Barbara, Santa Barbara, California 93106, United States

**Keywords:** targeted proteomics, liquid chromatography with tandem mass spectrometry (LC-MS/MS), signature peptides, sample preparation, method comparison

## Abstract

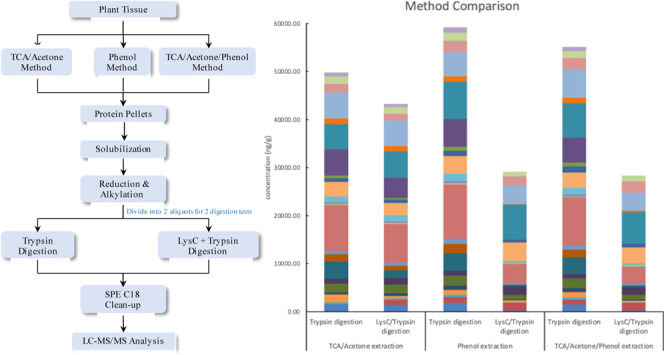

This study was conducted to optimize a targeted plant proteomics approach from signature peptide selection and liquid chromatography with tandem mass spectrometry (LC-MS/MS) analytical method development and optimization to sample preparation method optimization. Three typical protein extraction and precipitation methods, including trichloroacetic acid (TCA)/acetone method, phenol method, and TCA/acetone/phenol method, and two digestion methods, including trypsin digestion and LysC/trypsin digestion, were evaluated for selected proteins related to the impact of engineered nanomaterials (ENMs) on wheat (*Triticum aestivum*) plant growth. In addition, we compared two plant tissue homogenization methods: grinding freeze-dried tissue and fresh tissue into a fine powder using a mortar and pestle aided with liquid nitrogen. Wheat plants were grown under a 16 h photoperiod (light intensity 150 μmol·m^–2^·s^–1^) for 4 weeks at 22 °C with a relative humidity of 60% and were watered daily to maintain a 70–90% water content in the soil. Processed samples were analyzed with an optimized LC-MS/MS method. The concentration of selected signature peptides for the wheat proteins of interest indicated that the phenol extraction method using fresh plant tissue, coupled with trypsin digestion, was the best sample preparation method for the targeted proteomics study. Overall, the optimized approach yielded the highest total peptide concentration (68,831 ng/g, 2.4 times the lowest concentration) as well as higher signature peptide concentrations for most peptides (19 out of 28). In addition, three of the signature peptides could only be detected using the optimized approach. This study provides a workflow for optimizing targeted proteomics studies.

## 1 Introduction

Plant proteomics is a novel approach to generating knowledge about the proteins as biomarkers of the plant response to biotic and abiotic stresses.^1^ Particularly, modern mass spectrometry (MS)-based proteomics technologies, including nontargeted proteomics and targeted proteomics, have enabled the identification and quantification of the plant proteome that helps to understand the molecular mechanisms underlying plant phenotypes.^2^ Nontargeted proteomics is a discovery-based comprehensive analysis that quantifies thousands of proteins detectable in samples and is the most commonly used in plant proteomics.^2^ It is generally performed using data-dependent acquisition (DDA) with a quadrupole time-of-flight (Q-TOF) tandem mass spectrometer.^2,3^ However, the approach lacks accuracy and reproducibility due to the characteristics of a full-spectrum scan.^3,4^ The scans are performed over the full accessible mass range with the highest abundance ions selected as precursor ions for fragmentation. Since the selection of precursor ions is a stochastic process, DDA generates missing values and low reproducibility.^3,4^ Although nontargeted proteomics allows for the comprehensive analysis of proteins, the accuracy remains limited due to the broad-scale quantification.^3^ Since more than 100,000 peptides may be identified, it is impossible to develop calibration curves coupled with internal standards for them. Thus, the results of nontargeted proteomics are semiquantitative, reporting relative abundances rather than calibrated results.^5^ In contrast, targeted proteomics employs selected reaction monitoring (SRM) to analyze selected signature peptides in order to quantify the proteins of interest, leading to high sensitivity, accuracy, and reproducibility.^6,7^ Only the selected peptide precursor ion (Q1) with certain fragment ions (Q3) will be detected, since only specific mass-to-charge ratios (*m*/*z*) of Q1 and Q3 will be filtered into the detector.^6,8,9^ Calibration curves are developed for each peptide, with rigorous quality assurance. Therefore, targeted proteomics approaches can perform a specific, high-quality quantification of a limited set of preselected peptides for targeted proteins, which is useful for hypothesis-driven experiments.^4,10,11^ However, there is a need to optimize the methods used in targeted plant proteomics to ensure high reproducibility of results.

Several studies have employed targeted proteomics to determine allergen levels in plants such as soybean,^12^ hazelnut,^13^ wheat,^14^ and maize.^15^ Chawade et al. identified and analyzed potential protein biomarkers for potato plant breeding with targeted proteomics approaches, which leads to new possibilities of protein-based quantitation for understanding molecular mechanisms at the post-transcriptional level.^16^ Targeted proteomics was also used to characterize specific plant biological processes at the proteome level. Stecker et al. identified several regulatory proteins in *Arabidopsis* as specific targets for early events in dehydration responses and provided insights into plants’ biological processes involved in the osmotic stress response.^8^ Different methods of sample preparation and analysis were employed in these studies, but there was no detailed evaluation and optimization of the various steps in the analytical method.

To exemplify the use of proteomics in plant studies, we considered the exposure of crops to engineered nanomaterials (ENMs). ENMs have been studied for use in agriculture, especially as nanopesticides and nanofertilizers, to increase productivity.^17,18^ With the growing agricultural application of ENMs, exposure to ENMs as trending abiotic stress has drawn the attention of researchers to plant proteomics studies. Previous nontargeted proteomics studies have revealed plant responses to ENMs related to abiotic stress at the protein level (Table S1). For example, several studies investigated the proteomic response of *Oryza sativaL*,^19^*Triticum aestivum,*^20^ and *Glycine max*(21) after exposure to silver nanoparticles and identified responsive proteins that are involved in oxidative stress tolerance, electron transfer and signaling, transcription and protein degradation, and *N*-metabolism. The effects of cerium dioxide nanoparticles on *Phaseolus vulgaris* were also investigated with proteomic analysis, and the responsive proteins involved in oxidative stress regulation, photosynthesis and protein biosynthesis, and turnover were revealed.^22,23^ However, these qualitative results cannot fill the knowledge gap of the mechanisms underlying the biological responses to ENMs at the molecular level. By quantifying a specific set of ENM-responsive proteins with targeted proteomics, the changes in targeted proteins can provide clues about the perturbations in biological pathways triggered by ENMS,^24^ and hypotheses such as “the exposure of plants to metal-based ENM triggers defense responses in plant cells through specific biological pathways and affect protein regulation” can be tested.

Developing robust and specific assays for targeted plant proteomics can be challenging. First, it is important to choose targeted proteins that are relevant to the research hypothesis. Next, the signature peptides unique to those proteins need to be selected. The signature and isotopically labeled peptides selected as internal standards need to be synthesized to prepare analytical standards for liquid chromatography with tandem mass spectrometry (LC-MS/MS) method development. Then, an LC-MS/MS analytical method with high accuracy and sensitivity for the signature peptides and experimental design needs to be developed. Finally, the biggest challenge is to optimize sample preparation methods to extract the proteins of interest from plant tissue, followed by proteolytic digestion and peptide purification to achieve samples suitable for LC-MS/MS analysis. After completing these steps, the acquired data can finally be interpreted to accept or reject the research hypothesis. Currently, there is no published study that evaluates and optimizes these critical steps in targeted plant proteomics from beginning to end.

In this study, we optimized a targeted plant proteomics approach (Figure S1) for selected proteins related to the impact of ENMs on crop plant growth, using wheat as the crop of interest. First, signature peptides were selected and synthesized to order. Then, the LC-MS/MS analytical method for the selected peptides was optimized. Next, we evaluated 3 typical protein extraction and precipitation methods and 2 proteolytic digestion methods to develop the most effective sample preparation procedures for targeted plant proteomics. Finally, the finalized sample preparation method was used to process fresh and freeze-dried plant tissues to determine the best homogenization method. The optimized protocol for targeted proteomics in plant systems can serve as a template for food and plant researchers to perform targeted proteomics based on their specific research hypotheses.

## 2 Materials and Methods

### 2.1 Selection of Signature Peptides

For this study, 24 proteins were first selected as targets based on the reported importance for wheat growth and response to ENMs in previous nontargeted proteomics studies (Table S1). With the list of targeted proteins, signature peptides were selected based on a public wheat proteome database (wheatproteome.org) with the criteria discussed in Section 3.1. By searching for proteins within metabolic pathways of interest for testing the hypothesis, a list of potential signature peptides was generated. The wheat proteome database provided information on relative peptide abundance, whether the peptide is MRM-detectable, and the occurrence of this peptide sequence within the entire wheat proteome. If the peptide is only present in a particular protein, it is a signature peptide candidate. Considering the pathways and proteins identified in previous nontargeted studies, the peptides were filtered into a list of 28 signature peptide candidates (Table S1).

### 2.2 Materials

*T. aestivum* (wheat) seeds were purchased from Harmony Farms KS (Jennings, KS). Sodium hypochlorite solution, Triton X-100, protease inhibitor cocktail, dithiothreitol (DTT), iodoacetamide (IAA), trypsin protease, trifluoroethanol (TFE), formic acid, ammonium acetate, trichloroacetic acid (TCA), dimethyl sulfoxide (DMSO), 0.5 M pH 8.0 ethylenediaminetetra-acetic acid (EDTA), sucrose, high-performance liquid chromatography (HPLC) grade water, acetone, isopropyl alcohol (IPA), and methanol were obtained from Sigma-Aldrich (St. Louis, MO). Urea, ammonium bicarbonate, and acetonitrile (ACN) were purchased from Spectrum Chemicals (New Brunswick, NJ). Tris-buffered phenol solution, 1.5 M pH 8.8 Tris-HCl solution, LysC/trypsin protease mix, phenylmethanesulfonyl fluoride (PMSF), 2-mercaptoethanol (2-ME), sodium *n*-dodecyl sulfate (SDS), and 5 mL and 15 mL of the Eppendorf centrifuge tube were purchased from Fisher Scientific (Waltham, MA). The C18 cartridge (Waters Sep-Pak C18 1 cc, 50 mg of the sorbent) was purchased from Waters Corporation (Milford, MA).

The analytical standards of the 28 selected peptides (Table S1) were purchased from GenScript (Piscataway, NJ). These standards were synthesized as ordered in a white lyophilized powder phase with ≥95% HPLC purity. For each peptide, 1 mg/mL working stock solution was prepared by dissolving the standard powder into HPLC-grade water for water-soluble peptides (IQNGGTEVVEAK, SVHEPMQTGLK, TAVAAVPYGGAK, LVGVSEETTTGVK, VAEGDAEDVDRAVVAAR, KALDYEELNENVK, SGDVYIPR, GMAVPDSSSPYGVR, GNATVPAMEMTK, EFAPSIPEK, FVIGGPHGDAGLTGR, AADNIPGNLYSVK, TVVSIPNGPSELAVK, TLGELPAGSVIGSASLRR, YIGSLVGDFHR, TALIDEIAK, VAPEVIAEYTVR, IGGLTLNELGR, TLAEEVNQAFR, IGLFGGAGVGK, VQLLEIAQVPDEHVNEFK, KPWNLSFSFGR, and TWPEDVVPLQPVGR) or 50% (v:v) ACN in HPLC-grade water for non-water-soluble peptides (ADGGLWLLVR, TAIAIDTILNQK, FASINVENVEDNRR, VAEFSFR, and AAVIGDTIGDPLK). Peptide stock solutions were stored at −20 °C. Isotopically labeled peptide standards were also purchased from GenScript (Piscataway NJ) to use as an internal standard for LC-MS/MS analysis and quantitation. The selected internal standards include SVHEPMQTGLK{Lys(13C6,15N2)}, SGDVYIPR{Arg(13C6,15N4)}, TALIDEIAK{Lys(13C6,15N2)}, and KPWNLSFSFGR{Arg(13C6,15N4)}. A 1 mg/mL working stock solution for each internal standard was prepared in HPLC-grade water and stored at −20 °C.

### 2.3 LC-MS/MS Analysis Method

The working stock solution of 28 peptide standards and 4 isotopically labeled internal standards was diluted 100 times with water to reach a concentration of 10 μg/mL for compound optimization using an Agilent InfinityLab 1290 Infinity II Series liquid chromatography system coupled to an Agilent 6470 triple quadrupole mass spectrometer in positive ionization mode. Then, a mixture of all 28 peptides and 4 internal standards was prepared in 30% ACN with 0.1% formic acid and 3% DMSO in water at 1000 ng/mL to optimize the column and mobile phase to separate peaks of peptides with adequate abundance and sensitivity. An Agilent Polaris 3 C18-Ether column (150 mm × 3.0 mm, p/n: A2021150X030) coupled with a gradient mobile phase (A: Water + 0.1% (v:v) formic acid + 3% (v:v) DMSO; B: ACN + 0.1% (v:v) formic acid + 3% (v:v) DMSO) was selected as the optimal HPLC settings (Table S2). The flow rate was set to 0.4 mL/min with a column temperature of 25 °C and a 2 μL injection volume. The gradient mobile phase started at 5% B and gradually increased to 70% B in 10 min, then decreased to 5% B to re-equilibrate the column. Source optimization was performed by an agilent source optimizer to optimize MS settings (Table S2) including 340 °C gas temperature at a 12 L/min flow rate, 250 °C sheath gas temperature at a 9 L/min flow rate, nebulizer at 40 PSI, a capillary voltage of 3500 V, and a nozzle voltage at 2000 V. The total run time for each sample was 14 min. Needle wash with TFE was done between injections.

For each analyte, two pairs of transitions (*m*/*z* values associated with the precursor and fragment ions) with the highest abundance and signal-to-noise (s/n) ratio were selected for each compound as a quantifier and qualifier. The limit of detection (LOD) of each peptide was calculated by diluting standards until the concentration that gives a signal/noise = 3. The method detection limit (MDL) was calculated based on the sample extraction method. Since 200 mg of the plant tissue was extracted and reconstituted into 1 mL for instrument analysis, MDL (ng/g) = LOD (ng/mL)/0.2 (g/mL) = 5 × LOD (ng/g).

Calibration standards were prepared at 8 levels, including 1, 2.5, 5, 10, 25, 50, 75, and 100 ng/mL. 50 ng/mL of internal standards were added into each level of calibration standards and plant samples to adjust for matrix effects during quantification.

### 2.4 Plant Growth, Harvest, and Homogenization

As one of the most important crop plants, wheat (*T. aestivum*) was selected as the model plant for this research. This project focused on early-stage wheat plants since stressors at this stage may affect the formation of tillers that ensures the yield potential of wheat.^25^ Wheat plants were grown for 4 weeks to harvest the early-stage plant tissue for the experiments.

Before germination, all wheat seeds were sterilized in 1% sodium hypochlorite solution for 10 min., followed by 5 rinses with nanopure water. Then, sterilized seeds were soaked in nanopure water overnight before germination. Vermiculite was used as the growth matrix since it helps to maintain good aeration while simultaneously retaining water and nutrients that eventually are released for plant adsorption. Vermiculite was saturated with a 10% Hoagland solution and then transferred into plant pots up to 2.5 cm below the rim.^26^ Then, 80 soaked seeds were planted (4 seeds per pot) with tips facing up to ensure successful germination, then covered by vermiculite to fill the pot. Each pot was watered daily with 20 mL of 10% Hoagland water to maintain a 70–90% water content. Plants were grown under a 16 h photoperiod (light intensity 150 μmol·m^–2^·s^–1^) for 4 weeks at 22 °C with a relative humidity of 60%. A diluted 10% Hoagland solution was employed throughout the project to provide sufficient water and nutrients for plant growth.^26^ The concentrated Hoagland solution was prepared in nanopure water using 82.6 mg/L Ca(NO_3_)_2_·4H_2_O, 308.7 mg/L CaCl_2_·2H_2_O, 233.23 mg/L Mg(NO_3_)2·6H_2_O, 132 mg/L KH_2_PO_4_, 25.8 mg/L KNO_3_, 1.43 mg/L H_3_BO_3_, 4.04 mg/L Fe(NO_3_)3·9H_2_O, and 0.11 mg/L (Zn(NO_3_)_2_)·6H_2_O.^26^

After 28 days, the shoots of 80 wheat plants were harvested and divided into two parts, 40 plants in each group, to test different sample homogenization strategies. The first portion was ground into a fine powder directly, starting with fresh plant tissue frozen with liquid nitrogen and then ground with a ceramic mortar and pestle for homogenization. The second group was freeze-dried with lyophilizer (HRFDSSS Freeze Dryer, Harvest Right) and then finely ground into powder using a mortar and pestle with liquid nitrogen aided for homogenization. The two groups of homogenized plant tissue samples were stored at −80 °C until further processing and analysis.

### 2.5 Sample Preparation and Protein Digestion

To extract targeted peptides from plant samples, plant tissues were processed through protein extraction and precipitation, proteolytic digestion, and peptide purification. The general workflow starts with protein extraction from plant tissues using an extraction buffer, followed by protein precipitation to remove biological interferences from pigments, carbohydrates, nucleic acids, and other biomolecules using organic solvents such as acetone and methanol. Then, protein pellets are solubilized with a urea solution and processed through proteolytic digestion to cleave proteins into MRM-detectable peptide sequences. Finally, the digested peptides are purified via solid-phase extraction (SPE) before LC-MS/MS analysis.

**Figure 1:**
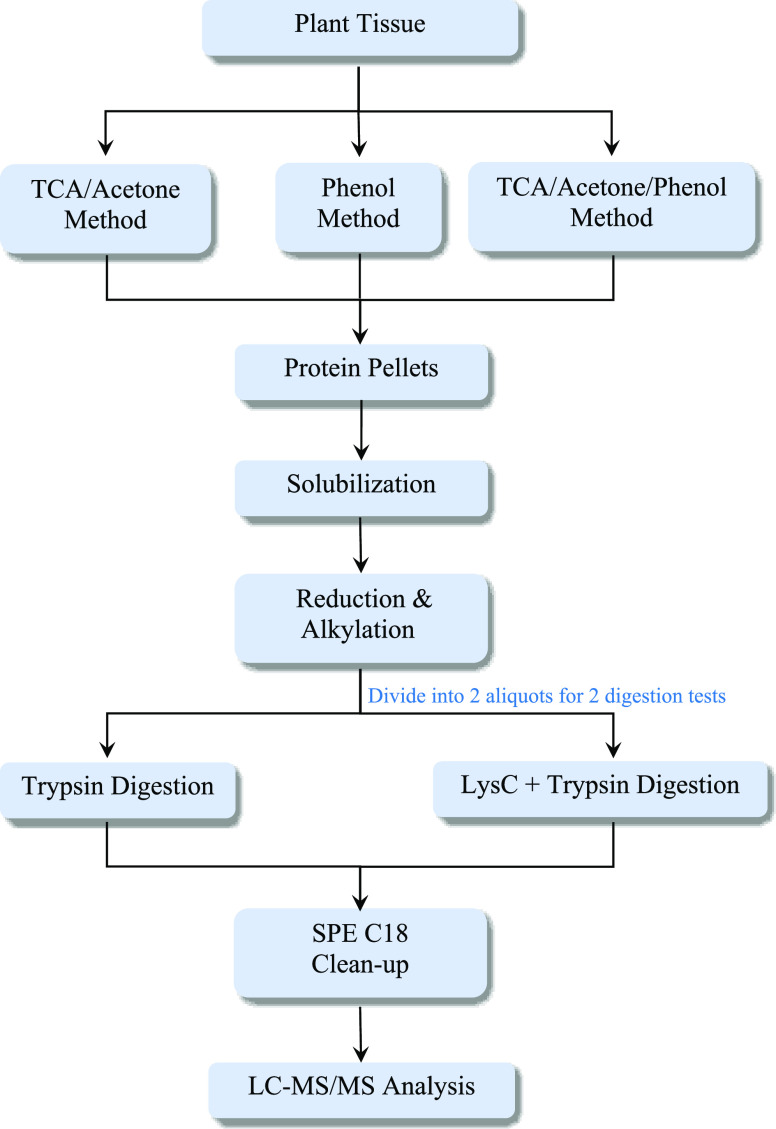
Flowchart of method comparisons of 3 protein extraction and precipitation methods, with 2 protein digestion methods.

To optimize the protein extraction and precipitation method, the most popular approaches including the TCA/acetone method,^27,28^ phenol method,^29^ and TCA/acetone/phenol method^30^ were compared in this study (Figure 1). In addition, two digestion methods, including trypsin digestion and LysC/trypsin digestion, were also compared. Homogenized fresh shoot tissues were used for these method comparisons. Full details of these methods are in the Supporting Information.

#### 2.5.1 Protein Extraction/Precipitation

**Figure 2:**
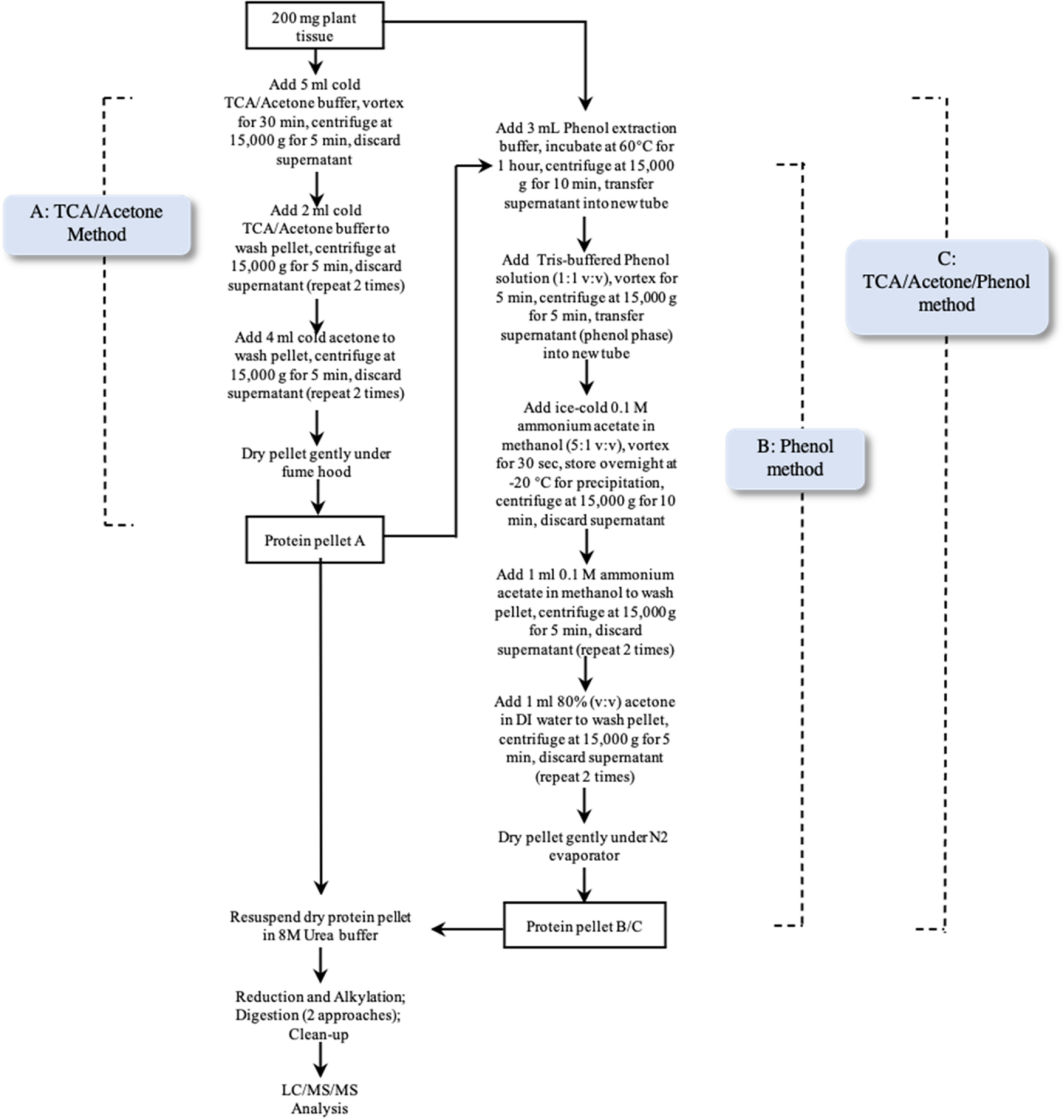
Flowchart of 3 protein extraction and precipitation methods, including the (A) TCA/acetone method, (B) phenol method, and (C) TCA/acetone/phenol method.

Two hundred mg of the plant sample was weighed out into a 5 mL centrifuge tube and processed with 3 methods of protein extraction and precipitation, including A: TCA/acetone method, B: phenol method, and C: TCA/acetone/phenol method, to achieve protein pellets (Figure 2). Full details of these 3 methods are in the Supporting Information. Procedures were modified from previous studies and are discussed in Section 3.2.1.

#### 2.5.2 Protein Digestion

Protein pellets A, B, and C, achieved as per Section 2.5.1, were reduced and alkylated with DTT and IAA. Then, the protein solution was divided into two aliquots to be digested with 2 digestion approaches, including trypsin digestion and LysC/trypsin digestion. Full details of protein reduction and alkylation and 2 protein digestion approaches are in the Supporting Information.

#### 2.5.3 Peptide Purification

Solid-phase extraction (SPE) cleanup with C18 cartridges (Waters Sep-Pak C18 1 cc, 50 mg of the sorbent) was used for peptide purification after protein digestion. Full details of peptide purification are in the Supporting Information.

### 2.6 Statistical Analysis

Three replicates were prepared for each method test. The average concentration of three sample replicates was calculated for each peptide to make method comparisons. Among compared extraction methods, the number of peptides showing the highest average concentration was counted, and the one with the highest number is considered to be the most efficient method. In addition, the total concentration of all 28 peptides was calculated for each method as another criterion to make the choice of the best method. Data were presented with a stacked column using Microsoft Excel to visualize the method comparisons.

## 3 Results and Discussion

### 3.1 Selection of Signature Peptides

The critical step to start a targeted proteomics project is the selection of the proteins that will serve to test the hypothesis and their corresponding “signature” peptides. For specific research hypotheses, the selection of proteins can be based on a preliminary nontargeted proteomic analysis, literature knowledge, and/or public data. With the list of targeted proteins, targeted peptides for quantification can be selected using either empirical proteomics data or prediction algorithms.^31^ Ideally, candidate peptides can be selected using MS data from in-house or public empirical data. This is the “gold standard” for targeted proteomics since the selected peptides have already been demonstrated to be present in the proteins of interest, cleavable, and detectable via MS.^31^ For this study, targeted proteins were selected based on literature review, and their signature peptides were selected based on the public database.

To assure a successful targeted proteomics assay, there are several criteria for selecting the targeted peptides. First, peptides need to be unique to the protein, which are denominated signature peptides, to enable the specificity of the analysis. Second, peptides must be detectable by MS since targeted proteomics utilizes MRM detection. Selection based on empirical MS data is more reliable than predictions. Additionally, to ensure a high response and stability of the signature peptides, criteria such as proper peptide length, hydropathy, reactive residues, and digestion parameters should be considered.^31^ Typically, the optimal peptide length for MRM detection is 7 to 20 amino acids, which is the typical length of tryptic peptides produced by trypsin digestion. In addition, reactive amino acid residues that could be modified during sample preparation should be avoided. Reactive residues that potentially lead to modifications include cysteine, methionine, and tryptophan (oxidation), n-terminal glutamine (pyroglutamic acid formation), asparagine, or glutamine, followed by glycine (deamidation) and aspartic acid, followed by glycine (dehydration), proline (peptide chain cleavage), and histidine (additional charge states).^31^ Additional criteria included high abundance of the protein and peptide and a short peptide length to reduce the cost of synthesis. Based on these criteria, 28 signature peptide candidates were selected (Table S1).

### 3.2 Optimization of LC-MS/MS Analysis for Selected Peptides

**Figure 3:**
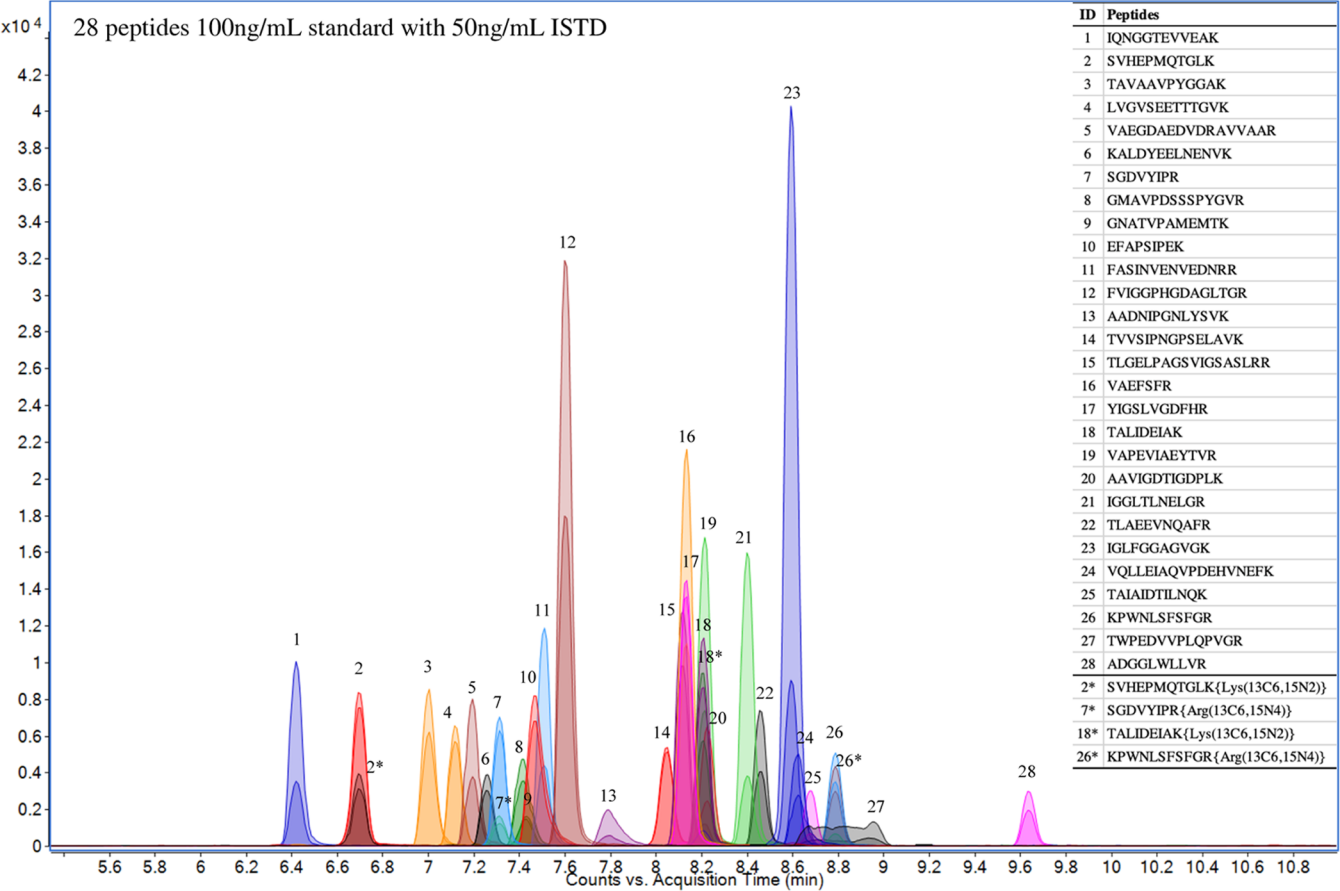
LC-MS/MS chromatograph of 28 peptides standards at 100 ng/mL with 50 ng/mL internal standards.

Figure 3 shows the LC-MS/MS chromatograph of 28 peptides standards (100 ng/mL with 50 ng/mL internal standard) using the optimized LC-MS/MS method. With optimized HPLC and MS conditions, the 28 peptides were separated well with great peak shape, which produced high signal-to-noise ratios and resulted in low LODs (Table 1). The retention time of the 28 peptides ranged from 6.4 to 9.6 min, and the 4 isotopically labeled internal standards eluted out at 6.7, 7.3, 8.2, and 8.8 min. An internal standard was selected for each of the 28 peptides based on the nearest retention time to adjust for matrix effects and ensure accurate quantitation.

**Table 1:** Transitions, LOD, and MDL for Each Peptide

				product ions						
ID	sequence	retention time (min)	precursor ion (*m*/*z*)	quant ion (*m*/*z*)	collision energy (V)	qual ion (*m*/*z*)	collision energy (V)	fragmentor (V)	LOD (ng/mL)	MDL (ng/g)
Peptides										
1	IQNGGTEVVEAK	6.42	623.2	242.1	20	86.1	32	132	0.02	0.08
2	SVHEPMQTGLK	6.70	409.8	110.2	40	84.0	40	96	0.41	2.05
3	TAVAAVPYGGAK	7.00	553.1	173.0	24	72.1	40	112	0.08	0.40
4	LVGVSEETTTGVK	7.12	660.7	86.0	36	72.1	40	137	0.09	0.44
5	VAEGDAEDVDRAVVAAR	7.19	582.0	786.9	16	72.0	40	96	0.16	0.81
6	KALDYEELNENVK	7.25	522.6	102.0	16	86.2	16	96	0.24	1.18
7	SGDVYIPR	7.31	454.0	548.3	16	60.1	40	96	0.01	0.04
8	GMAVPDSSSPYGVR	7.42	712.3	260.0	28	189.2	36	132	0.02	0.09
9	GNATVPAMEMTK	7.43	625.7	172.0	40	70.0	40	117	0.10	0.51
10	EFAPSIPEK	7.46	509.6	335.7	16	70.0	40	96	0.10	0.49
11	FASINVENVEDNRR	7.51	555.3	120.0	24	191.0	16	96	0.00	0.02
12	FVIGGPHGDAGLTGR	7.60	485.5	604.4	12	120.0	32	96	0.00	0.01
13	AADNIPGNLYSVK	7.79	681.8	877.4	20	230.0	32	127	0.08	0.38
14	TVVSIPNGPSELAVK	8.05	756.4	172.8	40	200.9	36	132	0.01	0.06
15	TLGELPAGSVIGSASLRR	8.12	595.7	635.9	16	186.9	20	117	0.00	0.02
16	VAEFSFR	8.13	428.5	171.0	12	72.1	24	96	0.01	0.03
17	YIGSLVGDFHR	8.13	422.1	494.3	8	86.0	28	96	0.01	0.03
18	TALIDEIAK	8.21	487.5	173.0	12	86.0	40	112	0.01	0.03
19	VAPEVIAEYTVR	8.21	674.3	589.0	16	70.0	40	147	0.06	0.28
20	AAVIGDTIGDPLK	8.23	635.7	72.0	32	86.0	32	132	0.06	0.30
21	IGGLTLNELGR	8.40	572.2	228.0	24	86.1	40	122	0.01	0.07
22	TLAEEVNQAFR	8.45	639.7	187.1	28	215.0	20	127	0.04	0.21
23	IGLFGGAGVGK	8.59	488.5	545.2	16	86.1	24	117	0.01	0.05
24	VQLLEIAQVPDEHVNEFK	8.62	703.8	227.8	24	72.1	36	142	0.01	0.06
25	TAIAIDTILNQK	8.68	651.3	173.1	24	86.0	40	112	0.10	0.50
26	KPWNLSFSFGR	8.79	670.3	84.0	36	70.1	40	137	1.17	5.84
27	TWPEDVVPLQPVGR	8.96	797.4	653.7	20	342.1	40	147	1.55	7.75
28	ADGGLWLLVR	9.63	550.7	159.0	40	86.0	40	117	0.02	0.11
Internal Standards										
2*	SVHEPMQTGLK{Lys(13C6,15N2)}	6.69	412.5	90.1	40	69.9	40	96		
7*	SGDVYIPR{Arg(13C6,15N4)}	7.31	459.0	558.3	12	260.0	16	91		
18*	TALIDEIAK{Lys(13C6,15N2)}	8.21	491.6	172.8	16	86.0	40	81		
26*	KPWNLSFSFGR{Arg(13C6,15N4)}	8.79	675.3	84.1	36	70.0	40	137		

To optimize HPLC conditions, different chromatography parameter settings including the mobile phase and sample solvent were compared to literature conditions. The parameters of this study and previous studies are listed in Table S3. Based on the literature review, reverse-phase columns with silica-based stationary phases such as octadecyl carbon chain (C18)-bonded silica were used to analyze peptides due to their strong affinity for compounds with a wide range of polarity. Ion-paring reagents such as TFA and formic acid in the mobile phase can help to deliver highly resolved separations of complex peptide mixtures from tryptic protein digests. In addition, trace amounts of DMSO (3–5%) in the mobile phase are also recommended for more efficient ionization and higher signal intensity of peptides.^32,33^ After testing several reversed-phase chromatography parameters from previous proteomics studies, the settings of this study were optimized to show the best peak shape and abundance for the targeted peptides.

During LC-MS/MS analysis method optimization, there was a carryover issue that resulted in peaks in solvent blanks immediately after an injection of the standard solution. This carryover issue can be caused by insufficient washing of the injection needle and valve of the autosampler since peptides can adsorb to HPLC components. For peptides containing hydrophobic residues, they can even be retained on HPLC columns despite the use of high concentrations of organic solvents for washing.^31^ The carryover issue can increase the variability of quantification and bias of analysis. In a previous study, Mitulovic et al. recommended the injection of TFE into the HPLC flow path and column to remove strongly bound peptides due to its properties to decoy peptides and ability to clean all parts of HPLC.^34^ In our study, we resolved the carryover issue by introducing an autosampler needle wash with 2 μL of TFE between injections.

### 3.3 Sample Preparation Optimization for Protein Extraction, Precipitation, and Digestion

**Figure 4:**
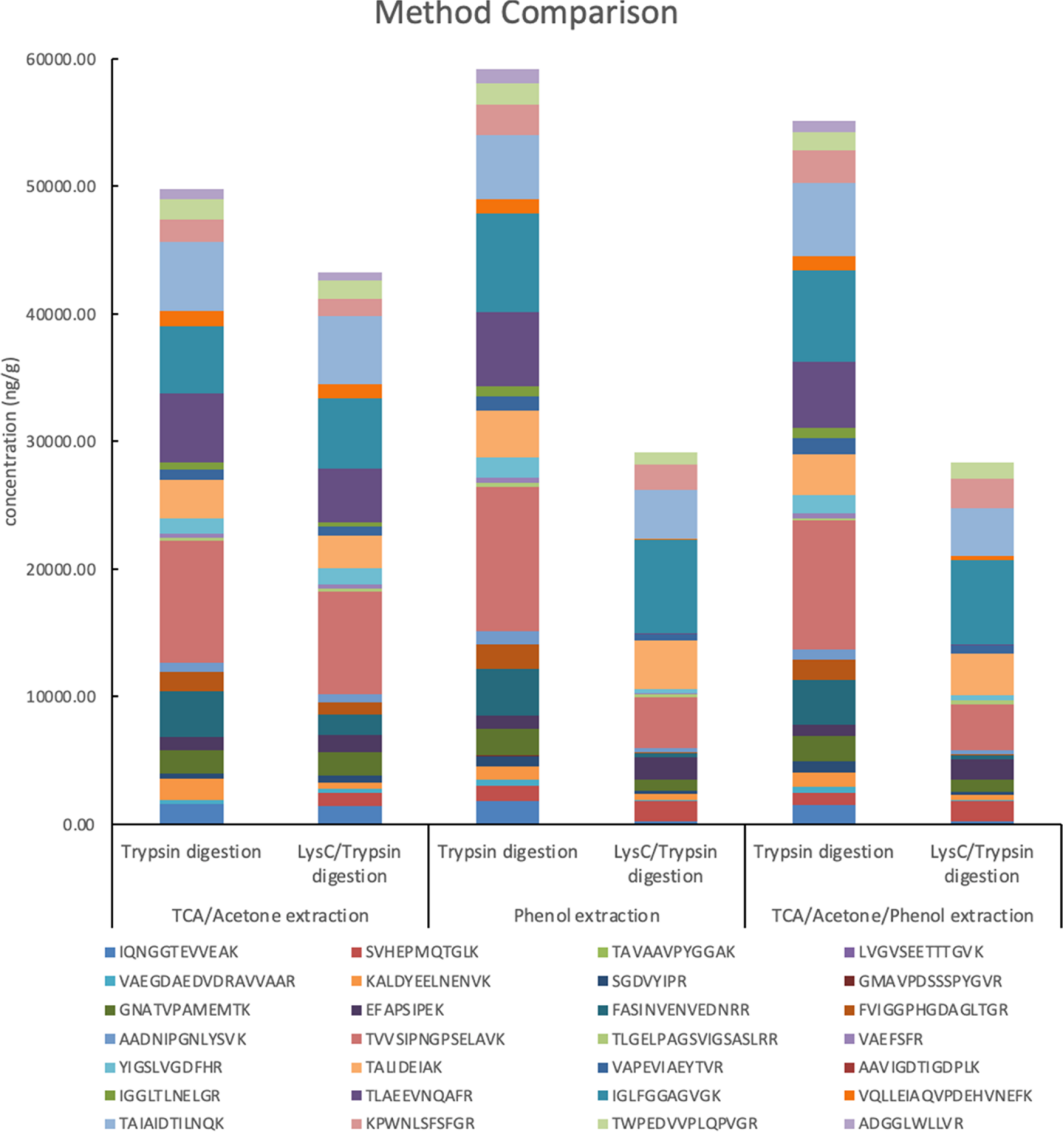
Peptide concentrations in plant tissues processed with 3 protein extraction and precipitation methods and 2 protein digestion methods.

Figure 4 presents the concentration of each targeted peptide in plant tissues processed with 3 protein extraction and precipitation methods and 2 protein digestion methods (full data in Table S4 in the Supporting Information). Three replicates were prepared for each test and the average concentrations were calculated. Among these 6 methods, the phenol method coupled with trypsin digestion yielded the highest concentration of most targeted peptides (17 out of 28), compared to the TCA/acetone/phenol method coupled with trypsin digestion (5 out of 28), the phenol method coupled with LysC/trypsin digestion (3 out of 28), the TCA/acetone method coupled with trypsin digestion (2 out of 28), the TCA/acetone/phenol method coupled with LysC/trypsin digestion (1 out of 28), and the TCA/acetone method coupled with LysC/trypsin digestion (0 out of 28). In addition, for the total peptide concentration (Figure 4), the phenol method coupled with trypsin digestion (59,193 ng/g) ranked highest, followed by the TCA/acetone/phenol method with trypsin digestion (55,107 ng/g), the TCA/acetone method with trypsin digestion (49,765 ng/g), the TCA/acetone method with LysC/trypsin digestion (43,263 ng/g), the phenol method with LysC/trypsin digestion (29,172 ng/g), and the TCA/acetone/phenol method with LysC/trypsin digestion (28,363 ng/g). Overall, trypsin digestion showed higher efficiency than LysC/trypsin digestion when coupled with any of the 3 extraction and precipitation methods. These results indicate that the phenol extraction method coupled with trypsin digestion is the best sample processing method for this study. The procedures of each method are discussed in the following sections.

#### 3.3.1 Protein Extraction and Precipitation

TCA/acetone-based precipitation methods are commonly used in plant proteomics since they involve a simple organic solution and limited steps. Damerval et al. originally developed this method that combines TCA and acetone precipitation,^35^ which can remove many compounds, particularly ions, lipids, pigments, phenolics, and terpenoids, from the samples more effectively than either TCA or acetone alone.^36^ This approach employs 10% TCA in acetone with 2-ME to precipitate proteins by adding the solution directly into the powdered plant tissue. The addition of 2-ME can unfold proteins and prevent the formation of disulfide bonds during precipitation, thus improving protein recovery.^28^ This less time-consuming and easier-to-operate precipitation method is recommended as a starting protocol for plant proteomic analyses and has been widely used in studies with minor modifications.^36^ However, the major drawback of this TCA/acetone precipitation approach is that protein pellets are very difficult to fully resolubilize. In the current study, an 8 M urea solution was used to resuspend protein pellets in an iced water bath with sonication. Around 1 h was needed to fully resolubilize the pellet. The difficulty of protein pellet solubilization from this method could result in the loss of targeted proteins.

A phenol extraction-based methanol precipitation method has also been widely applied in protein extraction from plants, especially for recalcitrant plant tissues.^29,36,37^ This method employs the solubility of proteins in phenol to partition the protein from the aqueous extraction buffer into the phenol phase and then precipitate the protein with ice-cold methanol with the addition of ammonium acetate. Isaacson et al. presented the phenol extraction-based methanol precipitation and the TCA/acetone precipitation methods as two protein extraction protocols successfully used with diverse plant tissues including tomato leaves and fruits, maize roots, and orange peels, some of which are recalcitrant tissues.^37^ Compared to the TCA/acetone method, the phenol method not only includes 2-ME as a reducing agent to prevent protein oxidation but also contains SDS to solubilize membrane-bound proteins, EDTA to inhibit metalloproteases and polyphenol oxidases, PMSF to irreversibly inhibit serine proteases, and protease inhibitors from preventing protein degradation. These added components may explain the increased recovery of protein using phenol extraction compared to the TCA/acetone method. In addition, sucrose in the buffer makes the aqueous phase heavier than Tris-buffered phenol, which facilitates separation by making the phenol phase buoyant. This liquid–liquid partitioning can extract protein from an aqueous buffer into the phenol phase and helps to clean up protein extract before protein precipitation, which can also lead to better protein recovery.

The TCA/acetone/phenol method integrating TCA/acetone precipitation and phenol extraction was developed by Wang et al. to utilize the advantages of both methods for optimized extraction.^30^ It starts with TCA/acetone precipitation, and then a phenol extraction buffer is used to resuspend protein pellets, followed by an aqueous buffer, phenol partition, and further protein precipitation using ammonium acetate in methanol. Although some nontargeted proteomics studies recommend this integrated method as an effective approach,^36,38,39^ that was not the case in the current study. Therefore, the simpler phenol extraction-based methanol precipitation method was used for sample analysis.

#### 3.3.2 Protein Digestion

Trypsin digestion is the “gold standard” for cleaving proteins into peptides for proteomics since it produces short peptides (0.6–1 kDa) with an ideal range for MS analysis (<3 kDa).^40^ Trypsin is also highly specific to cleave proteins at the carboxyl site of arginine and lysine residues, making these cleaved sites charged, which will be detectable by MS. However, for some tightly folded proteins, they are resistant to proteolytic digestion due to the inaccessibility of cleavage sites that are embedded in the structure. Predigestion with LysC before trypsin digestion can be implemented.^40,41^ This two-step digestion approach utilizes the characteristics of LysC, which shares lysine as a cleavage site with trypsin but has more tolerance to protein-denaturing reagents such as urea (8 M), in which trypsin is inactivated. Thus, LysC can first cleave protein into relatively long peptide sequences at the C-terminal of lysine in 8 M urea; then, trypsin can be activated to cleave the peptides further when urea is diluted below 2 M. Thus, LysC/trypsin can theoretically increase the digestion efficiency if there are a huge number of proteins to be digested, especially for nontargeted proteomics. However, for this targeted proteomics study, trypsin digestion proved to be the most effective for the targeted proteins and signature peptides and is also simpler.

#### 3.3.3 Peptide Purification

Peptide purification prior to LC-MS/MS analysis is a critical step to ensure the accuracy of peptide quantitation since it will remove contaminants that would interfere with LC-MS/MS analysis, such as salts from the extraction solution, reducing and alkylating reagents and trypsin from digestion.^42^ In the study by Majumdar et al., peptide solutions were desalted using Pierce C18 StageTips.^43^ By dispensing and aspirating the sample through a monolithic C18 reversed-phase sorbent, followed by elution with 0.1% formic acid in 50–95% ACN or methanol, C18 StageTips can effectively remove urea, salts, and other interfering contaminants before MS analysis. However, the small amount of sorbent can only bind up to 8 μg (10 μL tips) or 80 μg (100 μL tips) of total peptides. Instead, the peptide purification used in this study was solid-phase extraction (SPE) with C18 cartridges (Waters Sep-Pak C18 1 cc, 50 mg of the sorbent), as recommended by Mikołajczak et al. to purify protein digests with a retention–cleanup–elution strategy.^44^ The larger amount of sorbent and loading volume improves purification with a larger sample size (1–10 mL), yielding 1.7 mL of diluted peptide solution to be purified after protein digestion.

### 3.4 Fresh Tissue vs Freeze-dried Tissue

**Figure 5:**
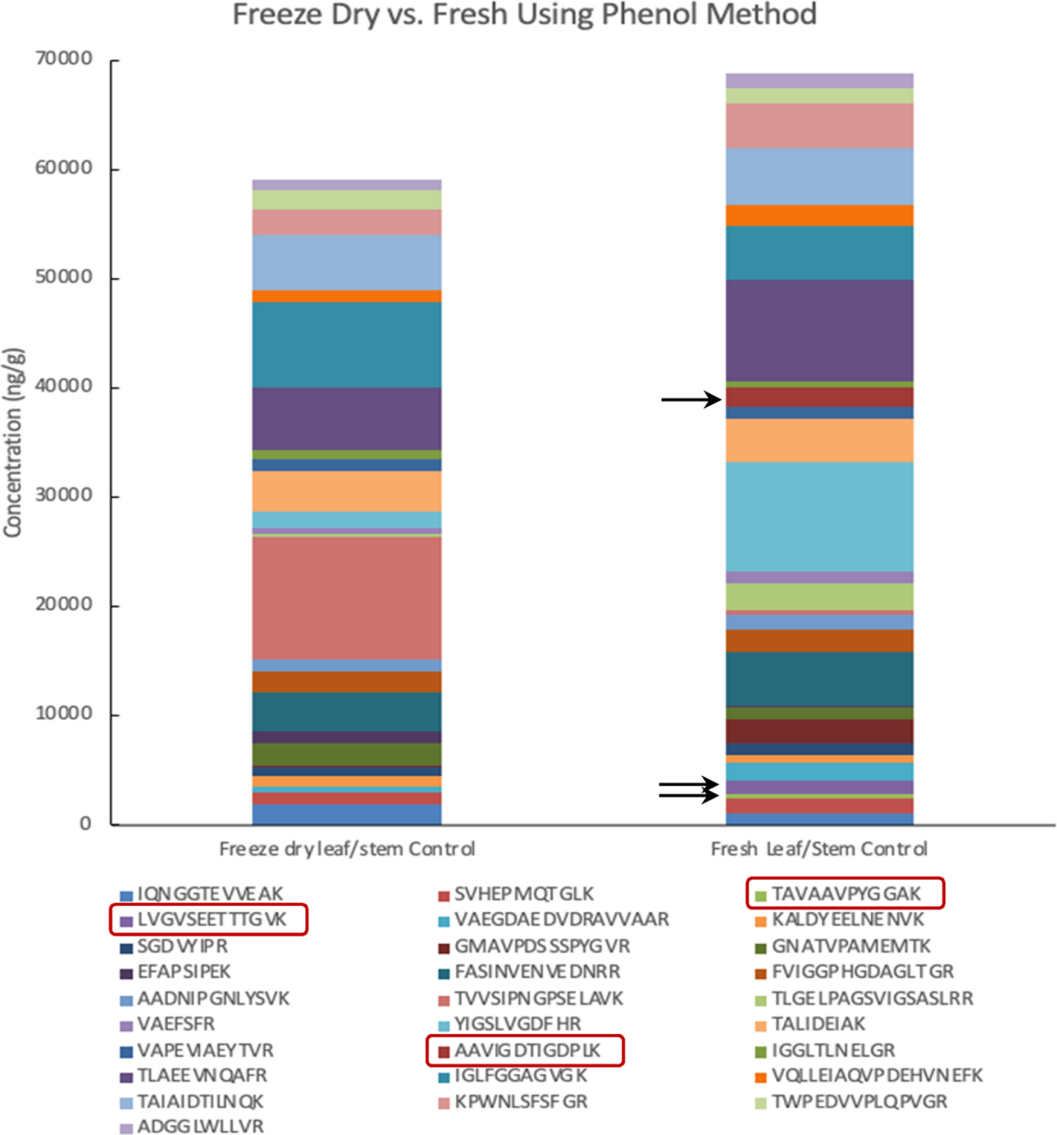
Peptide concentrations extracted from freeze-dried tissue vs fresh tissue.

To optimize the plant tissue homogenization method, both freeze-dried tissue and fresh tissue were processed using the optimized phenol extraction coupled with trypsin digestion. Three replicates were prepared for each test and the average concentrations were calculated. Full data are in Table S5 in the Supporting Information. A comparison of the total peptide and individual targeted peptide concentration extracted from freeze-dried tissue (59,193 ng/g) and fresh tissue (68,831 ng/g) indicated that it is better to use fresh tissue (Figure 5). In addition to a higher total peptide concentration, more peptides (19 out of 28) can be extracted from fresh wheat tissue than from freeze-dried tissue (9 out of 28) with higher concentrations. In particular, 3 peptides (i.e., TAVAAVPYGGAK, LVGVSEETTTGVK, and AAVIGDTIGDPLK) were only detectable in fresh tissue. Thus, the optimized homogenization method for this study was to grind fresh plant tissue into a fine powder using a mortar and pestle aided with liquid nitrogen.

## 4 Conclusions

In this study, an optimized workflow for targeted protein analysis was developed, starting from the selection of targeted proteins and signature peptides to test specific hypotheses concerning metabolomic pathways, followed by optimization of the extraction, digestion, and sample preparation methods. A comparison of 3 protein extraction and precipitation methods and 2 proteolytic digestion methods indicated that for the wheat proteins of interest, the phenol extraction method using fresh plant tissue, coupled with trypsin digestion, was the best sample preparation method for a targeted proteomics study. Overall, the optimized approach yielded the highest total peptide concentration as well as higher signature peptide concentrations for most peptides (19 out of 28). Three of the signature peptides could only be detected using the optimized approach. Since different plant tissues, or targeted proteins and signature peptides, may be preferentially extracted and digested by other methods, the workflow provides a template for optimizing targeted proteomics for other plant or food samples. Targeted proteomics techniques can also integrate with targeted metabolomics and genomics to provide a more comprehensive understanding of the plant response to biotic or abiotic stresses in the plant research field.

## Supporting Information Available

Detailed information on the procedures of three methods of protein extraction and precipitation, protein reduction and alkylation, two protein digestion approaches, and peptide purification; presented full data of peptide concentrations in plant tissues processed with 3 protein extraction and precipitation methods and 2 protein digestion methods and full data of peptide concentrations of each peptide extracted from freeze-dried tissue and fresh tissue; presented workflow of a targeted plant proteomics research; listed 28 signature peptides selected for the 24 targeted proteins based on literature reviews; presented HPLC and MS conditions of the optimized LC-MS/MS analysis method; and summarized chromatography parameters of this study versus previous plant proteomics studies (PDF)
