# Supplementary material for: Optimization of Targeted Plant Proteomics Using Liquid Chromatography with Tandem Mass Spectrometry (LC-MS/MS)
Source: ACS Agric Sci Technol. 2023 Apr 17;3(5):421–31. doi: 10.1021/acsagscitech.3c00017 (PMC10189723; doi:10.1021/acsagscitech.3c00017)
Supplement: Supplementary file 1 — as3c00017_si_001.pdf [file as3c00017_si_001.pdf]

Supporting Information for

Optimization of targeted plant proteomics using liquid chromatography with tandem mass spectrometry (LC-MS/MS)

Weiwei Li and Arturo A. Keller*

Bren School of Environmental Science and Management, University of California at Santa Barbara, Santa Barbara, California 93106, USA

*Corresponding author: Tel: +1 805 893 7548; Fax: +1 805 893 7612. Email address: arturokeller@ucsb.edu

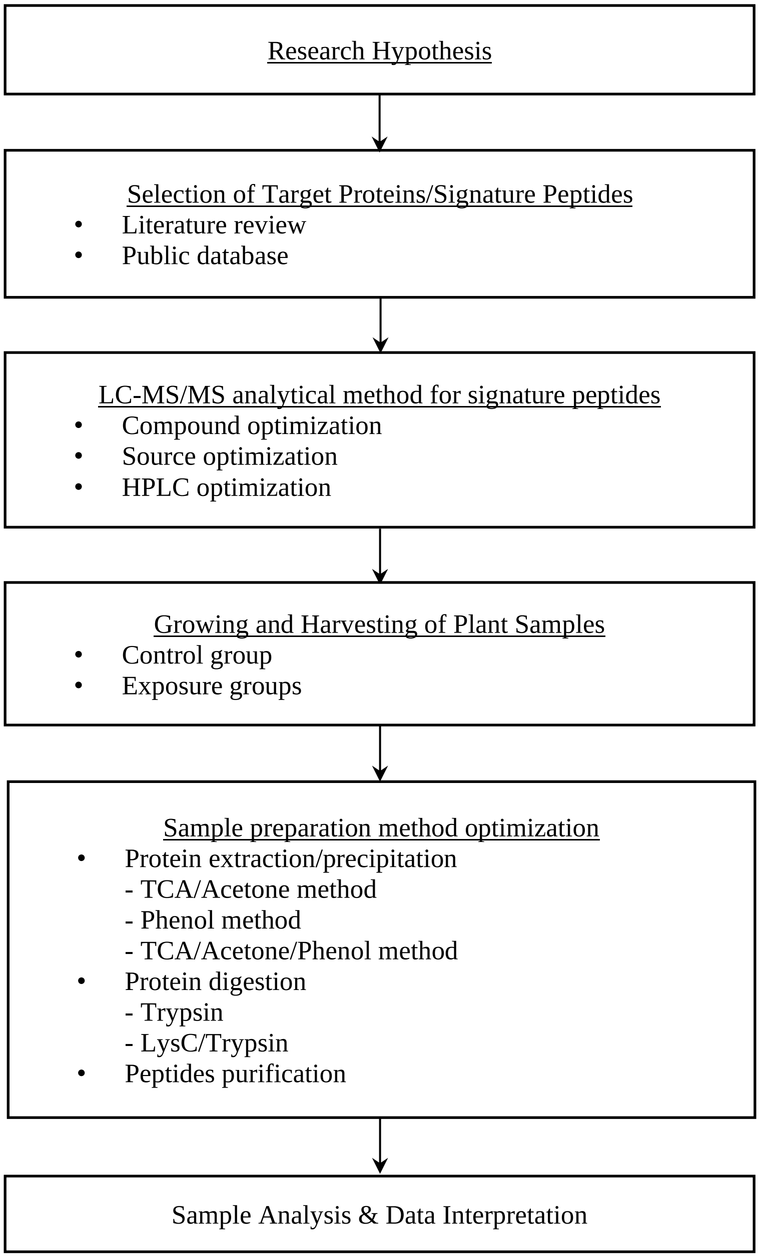

Figure S1. Workflow of a targeted plant proteomics research

Three methods of protein extraction and precipitation

A) TCA/Acetone Method:

A 200 mg plant tissue sample was mixed with 5 mL of cold TCA/acetone buffer by vortexing for 30 min at 4 °C, followed by centrifuging at 15,000 g for 5 min, with supernatant discarded. An additional 2 ml of cold TCA/acetone buffer was then added into the tube to wash the pellet, followed by centrifuging at 15,000 g for 5 min with supernatant discarded after each centrifugation (repeated 2 times). Then 4 ml of cold acetone was added into tube to wash TCA

off, followed by centrifuging at 15,000 g for 5 min with supernatant discarded (repeated 2 times). The protein pellet A was dried under the fume hood overnight at room temperature.

B) Phenol Method:
A 200 mg plant tissue sample was mixed with 3 mL phenol extraction buffer, then incubated at 60°C for 1 hour, followed by centrifuging at 15,000 g for 10 min. Then, 3 mL of supernatant was transferred into a new 15 mL centrifuge tube and mixed with 2.5 mL of phenol solution (Tris-buffered) by vortexing for 5 min, followed by centrifuging at 15,000 g for 5 min. Then the phenol phase (supernatant) was transferred into a new 15 mL tube and mixed with 12.5 mL of ice-cold 0.1 M ammonium acetate in methanol by vortexing for 30 sec, and then stored overnight at -20 °C for protein precipitation, followed by centrifuging at 15,000 g for 10 min with supernatant discarded the next day. 1 ml of 0.1 M ammonium acetate in methanol was added into tube to wash pellet, followed by centrifuging at 15,000 g for 5 min with supernatant discarded (repeated 2 times). Then 1 ml of cold 80% (v:v) acetone in DI water was added into the tube to wash the protein pellet and remove phenol, methanol and ammonium acetate, followed by centrifuging at 15,000 g for 5 min with supernatant discarded (repeat 2 times). Then protein pellet B was dried gently under N_2 evaporator at room temperature.

C) TCA/Acetone/Phenol Method:
A 200 mg plant tissue sample was processed following the procedure of TCA/Acetone Method to achieve protein pellet A. Then the protein pellet A was resuspended with phenol extraction buffer and processed following procedure of phenol method to produce protein pellet C.

Extraction buffers used for 3 protein extraction/precipitation methods were prepared as following recipe:

TCA/Acetone buffer: 10% (v:v) TCA in acetone with 2% (v:v) 2-ME.
Dissolve 50 g of TCA in 400 mL of acetone. Bring the volume to 500 mL by adding acetone. Keep the solution at − 20 °C. Add 2% (v:v) 2-ME just before use.

Phenol extraction buffer: 1% (w:v) SDS, 0.15 M Tris-HCl (pH 8.8), 2% (v:v) 2-ME, 1 mM EDTA, 2 mM PMSF, 1% (v:v) protease inhibitors and 0.7 M sucrose.
Prepare buffer by mixing SDS (1%, w:v), Tris-HCl (pH 8.8, 0.15 M), EDTA (1 mM) and sucrose (0.7 M) in water and store at 4 °C. Prepare stock of PMSF (100mM) by dissolving 174 mg of PMSF in 10 mL of IPA and store at − 20 °C. Add 2% (v:v) 2-ME, 2 mM PMSF and 1% (v:v) protease inhibitors just before use.

Protein reduction and alkylation

Protein pellets A, B & C achieved after protein extraction and precipitation using 3 methods were resuspended and solubilized with 8 M urea, 50 mM ammonium bicarbonate solution. Then protein in solution was reduced and alkylated by the following steps: 1) add 5 mM DTT and incubate at 56 °C for 30 min with rotation; 2) add 20 mM IAA and incubate at room temperature for 30 min in dark; 3) add additional 20 mM DTT and incubate at room temperature for 30 min to consume unreacted IAA.

Two protein digestion approaches

1) Trypsin digestion

Protein solution was diluted with 50 mM ammonium bicarbonate to reduce the concentration of urea from 8 M to 1M. Then 2 μg of trypsin enzyme was added into solution and incubated overnight at 37 °C with rotation.

2) LysC/Trypsin digestion

2 μg of LysC/trypsin enzyme was added into protein solution and incubated at 37 °C for 4 hr with rotation. Then solution was diluted with 50 mM ammonium bicarbonate to reduce the concentration of urea from 8 M to 1M, followed by incubation overnight at 37 °C with rotation.

Protein digestion was stopped by adding formic acid into solution to reach a final concentration of 5% (v:v) the next day.

Peptide purification

Firstly, the cartridge was conditioned with 1 mL of 80% ACN with 5% formic acid in water. Then the cartridge was re-equilibrated with 1 mL of 5% formic acid, followed by loading digested peptide solution from 2.5.2. After sample loading, the cartridge was washed with 1.5 mL of 5% formic acid. Then peptides adsorbed onto the sorbent was eluted with 1.5 mL of 80 % ACN with 5% formic acid. The eluate was dried gently under N_2 evaporator at room temperature and then reconstituted to 30% ACN in water with 5% formic acid and 3% DMSO for LC-MS/MS analysis.

Table S1. List of 28 signature peptides for the 24 targeted proteins for this project.

Pathway	Protein	Peptides	Up or Down Regulation	Plant Species	Reference
Amino acid metabolism	AA degradation methionine	LVGVSEETTTGVK		Triticum aestivum	(1)
	AA synthesis methionine	GNATVPAMEMTK		Triticum aestivum	(1)
	S-adenosylmethionine synthase	FVIGGPHGDAGLTGR	+(Ag-NP)	Triticum aestivum	(2)
Fermentation	aldehyde dehydrogenase	VAEGDAEDVDRAVVAAR		Triticum aestivum	(1)
Glycolysis	glycolysis cytosolic branch UGPase	FASINVENVEDNRR		Triticum aestivum	(1)
	glycolysis cytosolic branch aldolase	VQLLEIAQVPDEHVNEFK		Triticum aestivum	(1)
H+ transporting pyrophosphatase	transport H+ transporting pyrophosphatase	AAVIGDTIGDPLK		Triticum aestivum	(1)
Hormone metabolism	lipoxygenase	GMAVPDSSSPYGVR	+(Al2O3-NP); -(Zn-NP, Ag-NP)	Glycine max	(3)
Mitochondrial electron transport / ATP synthesis	transport p- and v-ATPase	SGDVYIPR		Triticum aestivum	(1)
	ATP synthase delta chain	TALIDEIAK		Triticum aestivum	(1)
	ATP synthase beta subunit	IGLFGGAGVGK		Triticum aestivum	(1)
	ATP synthase F1-ATPase	TAIAIDTILNQK		Triticum aestivum	(1)
		SVHEPMQTGLK			
N-metabolism	glutamate dehydrogenase	TAVAAVPYGGAK	+(Al2O3-NP); -(Zn-NP, Ag-NP)	Glycine max	(3)

	glutamate synthase ferredoxin dependent	IGGLTLNELGR		Triticum aestivum	(1)
Photorespiratory pathway	aminotransferases peroxisomal	KALDYEELNENVK		Triticum aestivum	(1)
	photosystem II stability/assembly factor HCF136	AADNIPGNLYSVK	-(Ag-NP)	Triticum aestivum	(2)
		ADGGLWLLVR			
Photosynthesis / Calvin Cycle	calvin cycle aldolase	TVVSIPNGPSELAVK		Triticum aestivum	(1)
	calvin cycle FBPase	YIGSLVGDFHR		Triticum aestivum	(1)
	fructose-bisphosphate aldolase	VAPEVIAEYTVR	+(Ag-NP)	Triticum aestivum	(2)
		KPWNLSFSFGR			
	calvin cycle GAP	TLAEEVNQAFR		Triticum aestivum	(1)
Redox	dismutases and catalases	TWPEDVVPLQPVGR		Triticum aestivum	(1)
TCA / org transformation	malate dehydrogenase	EFAPSIPEK	+(Ag-NP)	Triticum aestivum	(2)
		IQNGGTEVVEAK			
	TCA aconitase	VAEFSFR		Triticum aestivum	(1)
Tetrapyrrole biosynthesis	tetrapyrrole synthesis prophobilinogen deaminase	TLGELPAGSVIGSASLRR		Triticum aestivum	(1)

Table S2. HPLC conditions and MS conditions for LC-MS/MS analysis method.

**HPLC Conditions**		
Column	Agilent Polaris 3 C18-Ether 150x3.0mm (p/n:A2021150X030)	
Mobile phase A	Water + 0.1% (v:v) formic acid +3% (v:v) DMSO	
Mobile phase B	Acetonitrile + 0.1% (v:v) formic acid +3% (v:v) DMSO	
Flow rate	0.40 mL/min	
Column temperature	25 °C	
Injection volume	2 μL	
Total run time	14 minutes	
Gradient	Time (min)	%B
	0.00	5
	10.00	70
	10.01	5
	14.00	5
**MS Conditions**		
Ionization mode	ESI Positive	
Gas temperature	340 °C	
Gas flow	12 L/min	
Nebulizer	40 psi	
Sheath gas temperature	250 °C	
Sheath gas flow	9 L/min	

Capillary voltage	Positive	Negative
	3,500 V	3,500 V
Nozzle voltage	Positive	Negative
	2,000 V	2,000 V

Table S3. Chromatography parameters of this study and previous plant proteomics studies

Column	Mobile Phase	Sample Solvent	Gradient	Reference
Agilent Polaris 3 C18-Ether 150 × 3.0mm column	A: 0.1% formic acid and 3% DMSO in water<br>B: 0.1% formic acid and 3% DMSO in ACN	30% ACN with 5% formic acid and 3% DMSO in water	5-70% B	This study
Polaris Ether 3-lm C18 2 × 250 mm column	A: 0.1% aqueous TFA<br>B: 90% ACN and 0.085% aqueous TFA	0.1% formic acid in water	0-50% B	([4])
in house made 75 µm × 40 cm, Reprosil-Gold C18, 3 µm resin, Dr. Maisch, Ammerbuch, Germany	A: 0.1% formic acid and 5% DMSO in water<br>B: 0.1% formic acid and 5% DMSO in ACN	0.1% formic acid in water	4-32% B	([5])
in-house made 17 cm fused silica capillary column (100 µm ID) packed with 3 µm Reprosil-C18 reverse phase material	A: 0.1% formic acid in water<br>B: 0.1% formic acid in 90% ACN	0.1% formic acid in water	0-34% B	([6])
Acclaim Pepmap C18 2 µm 100A, 75 µm i.d. × 15 cm length, Thermo-Fisher Scientific/Dionex	A: 0.1% formic acid in water<br>B: 0.08% formic acid in 90% ACN	1% formic acid in water	4-70% B	([7])
C18 high-capacity nano LC chip	A: 0.1% formic acid in water<br>B: 0.1% formic acid in ACN	2% ACN and 0.1% formic acid in water	2-100% B	([8])
cHiPLC nanoflex microfluidic C18 column 75 mm, 120 Å	A: 0.1% formic acid in water<br>B: 0.1% formic acid in ACN	0.5% formic acid in water	2-90% B	([9])
Acclaim PepMap C18 2 µm 100A, 75 µm i.d. × 15 cm length, Thermo-Fisher Scientific/Dionex	A: 0.1% formic acid in water<br>B: 0.08% formic acid in 90% ACN	1% formic acid in water	4-70% B	([10])
Polaris-HR-Chip-3 C18 column	A: 0.1% formic acid in water<br>B: 0.1% formic acid in ACN	5% ACN and 0.1% formic acid in water	5-35% B	([1])

Agilent Zorbax Eclipse Plus C18 RRHD 2.1 × 150 mm, 1.8 µm pore size column	A: 0.1% formic acid in water<br>B: 0.1% formic acid in 98% ACN	0.1% formic acid in water	3-97% B	([11])
YMC-Triart C18 column pore 12 nm, particle 3 µm, 150 mm length × 0.3 mm id column	A: 0.1% formic acid in water<br>B: 0.1% formic acid in ACN	97% ACN and 0.1% formic acid in water	3-80% B	([12])
in-house made 75 µm I.D. × 400 mm, 1.9 µm beads C18 Reprosil-HD, Dr. Maisch	A: 0.1% formic acid in water<br>B: 0.1% formic acid in 80% ACN	2% ACN and 0.1% formic acid in water	2-56% B	([13])
C18 reversed phase (3 uM, 100A pores, Dr. Maisch GmbH) column, packed in-house with 100uM ID and 18cm resin	A: 3%DMSO in water<br>B: 3% DMSO in ACN	5% formic acid in water	N/A	([14])

Table S4. Peptide concentrations in plant tissues processed with 3 protein extraction and precipitation methods and 2 protein digestion methods.

Peptides (ng/g)	TCA/Acetone Method		Phenol Method		TCA/Acetone/Phenol Method	
	Trypsin digestion	LysC/Trypsin digestion	Trypsin digestion	LysC/Trypsin digestion	Trypsin digestion	LysC/Trypsin digestion
IQNGGTEVVEAK	1577.37	1444.96	1846.93	246.42	1512.87	270.10
SVHEPMQTGLK	0	989.08	1149.65	1620.80	953.50	1547.17
VAEGDAEDVDRAVVAAR	298.85	335.04	491.87	37.68	473.86	89.71
KALDYEELNENVK	1668.41	519.91	1070.43	518.47	1145.45	423.94
SGDVYIPR	449.93	533.46	808.05	163.63	819.90	178.36
GMAVPDSSSPYGVR	0	0	58.72	0	30.97	0
GNATVPAMEMTK	1823.71	1804.15	2048.48	950.98	2012.05	992.22
EFAPSIPEK	1035.12	1366.18	1082.65	1724.25	851.83	1559.58
FASINVENVEDNRR	3581.39	1646.80	3618.37	334.73	3481.21	357.26
FVIGGPHGDAGLTGR	1487.53	955.47	1956.70	62.06	1614.19	86.57
AADNIPGNLYSVK	734.36	607.10	1013.28	278.35	777.54	309.76
TVVSIPNGPSELAVK	9602.24	8000.96	11322.80	4038.47	10144.39	3598.23
TLGELPAGSVIGSASLRR	205.70	261.86	272.85	224.88	177.34	299.89
VAEFSFR	328.27	314.13	417.83	44.42	397.22	0
YIGSLVGDFHR	1146.68	1284.12	1595.43	314.13	1441.63	419.95
TALIDEIAK	3075.50	2599.95	3643.72	3860.42	3133.17	3267.69
VAPEVIAEYTVR	753.35	650.46	1163.97	474.75	1340.03	645.14
IGGLTLNELGR	560.63	336.28	775.57	0	783.65	0
TLAEEVNQAFR	5472.09	4238.38	5793.60	54.12	5195.69	89.67
IGLFGGAGVGK	5202.16	5522.34	7720.47	7338.16	7112.62	6580.29

VQLLEIAQVPDEHVNEFK	1202.93	1112.87	1175.23	93.47	1106.21	351.78
TAIAIDTILNQK	5475.48	5298.47	5015.57	3808.02	5779.68	3691.17
KPWNLSFSFGR	1711.02	1400.37	2380.61	2021.94	2570.85	2364.00
TWPEDVVPLQPVGR	1582.58	1376.84	1697.58	962.01	1430.35	1240.71
ADGGLWLLVR	789.33	664.27	1072.36	0	820.98	0
**Total**	**49764.61**	**43263.46**	**59192.73**	**29172.15**	**55107.17**	**28363.19**

Table S5. Peptide concentration of each peptide extracted from freeze-dried tissue and fresh tissue.

**Peptides (ng/g)**	**Freeze-dried tissue**	**Fresh tissue**
IQNGGTEVVEAK	1846.93	1047.84
SVHEPMQTGLK	1149.65	1340.42
TAVAAVPYGGAK	0.00	464.67
LVGVSEETTTGVK	0.00	1224.88
VAEGDAEDVDRAVVAAR	491.87	1596.60
KALDYEELNENVK	1070.43	741.79
SGDVYIPR	808.05	1102.14
GMAVPDSSSPYGVR	58.72	2239.22
GNATVPAMEMTK	2048.48	1082.04
EFAPSIPEK	1082.65	121.58
FASINVENVEDNRR	3618.37	4900.71
FVIGGPHGDAGLTGR	1956.70	2028.29
AADNIPGNLYSVK	1013.28	1404.18
TVVSIPNGPSELAVK	11322.80	438.37
TLGELPAGSVIGSASLRR	272.85	2424.43
VAEFSFR	417.83	1029.86
YIGSLVGDFHR	1595.43	10050.64
TALIDEIAK	3643.72	4036.61
VAPEVIAEYTVR	1163.97	1029.60
AAVIGDTIGDPLK	0.00	1755.72
IGGLTLNELGR	775.57	611.32
TLAEEVNQAFR	5793.60	9221.31
IGLFGGAGVGK	7720.47	5029.47
VQLLEIAQVPDEHVNEFK	1175.23	1861.52
TAIAIDTILNQK	5015.57	5273.86
KPWNLSFSFGR	2380.61	4039.33
TWPEDVVPLQPVGR	1697.58	1330.47
ADGGLWLLVR	1072.36	1404.00
**Total**	**59192.73**	**68830.85**

(1) Duncan, O.; Trösch, J.; Fenske, R.; Taylor, N. L.; Millar, A. H. Resource: Mapping the *Triticum Aestivum* Proteome. *Plant J.* **2017**, *89* (3), 601–616. https://doi.org/10.1111/tpj.13402.
(2) Vannini, C.; Domingo, G.; Onelli, E.; De Mattia, F.; Bruni, I.; Marsoni, M.; Bracale, M. Phytotoxic and Genotoxic Effects of Silver Nanoparticles Exposure on Germinating Wheat Seedlings. *J. Plant Physiol.* **2014**, *171* (13), 1142–1148. https://doi.org/10.1016/j.jplph.2014.05.002.
(3) Hossain, Z.; Mustafa, G.; Sakata, K.; Komatsu, S. Insights into the Proteomic Response of Soybean towards Al 2 O 3 , ZnO, and Ag Nanoparticles Stress. *J. Hazard. Mater.* **2016**, *304*, 291–305. https://doi.org/10.1016/j.jhazmat.2015.10.071.
(4) Ren, D.; Pipes, G. D.; Liu, D.; Shih, L.-Y.; Nichols, A. C.; Treuheit, M. J.; Brems, D. N.; Bondarenko, P. V. An Improved Trypsin Digestion Method Minimizes Digestion-Induced Modifications on Proteins. *Anal. Biochem.* **2009**, *392* (1), 12–21. https://doi.org/10.1016/j.ab.2009.05.018.
(5) Hahne, H.; Pachl, F.; Ruprecht, B.; Maier, S. K.; Klaeger, S.; Helm, D.; Médard, G.; Wilm, M.; Lemeer, S.; Kuster, B. DMSO Enhances Electrospray Response, Boosting Sensitivity of Proteomic Experiments. *Nat. Methods* **2013**, *10* (10), 989–991. https://doi.org/10.1038/nmeth.2610.
(6) León, I. R.; Schwämmle, V.; Jensen, O. N.; Sprenger, R. R. Quantitative Assessment of In-Solution Digestion Efficiency Identifies Optimal Protocols for Unbiased Protein Analysis. *Mol. Cell. Proteomics MCP* **2013**, *12* (10), 2992–3005. https://doi.org/10.1074/mcp.M112.025585.
(7) Suliman, M.; Chateigner-Boutin, A.-L.; Francin-Allami, M.; Partier, A.; Bouchet, B.; Salse, J.; Pont, C.; Marion, J.; Rogniaux, H.; Tessier, D.; Guillon, F.; Larré, C. Identification of Glycosyltransferases Involved in Cell Wall Synthesis of Wheat Endosperm. *J. Proteomics* **2013**, *78*, 508–521. https://doi.org/10.1016/j.jprot.2012.10.021.
(8) Nelson, C. J.; Alexova, R.; Jacoby, R. P.; Millar, A. H. Proteins with High Turnover Rate in Barley Leaves Estimated by Proteome Analysis Combined with in Planta Isotope Labeling. *Plant Physiol.* **2014**, *166* (1), 91–108. https://doi.org/10.1104/pp.114.243014.
(9) Stecker, K. E.; Minkoff, B. B.; Sussman, M. R. Phosphoproteomic Analyses Reveal Early Signaling Events in the Osmotic Stress Response. *Plant Physiol.* **2014**, *165* (3), 1171–1187. https://doi.org/10.1104/pp.114.238816.
(10) Rogniaux, H.; Pavlovic, M.; Lupi, R.; Lollier, V.; Joint, M.; Mameri, H.; Denery, S.; Larré, C. Allergen Relative Abundance in Several Wheat Varieties as Revealed via a Targeted Quantitative Approach Using MS. *PROTEOMICS* **2015**, *15* (10), 1736–1745. https://doi.org/10.1002/pmic.201400416.
(11) Mikołajczak, B.; Fornal, E.; Montowska, M. LC–Q–TOF–MS/MS Identification of Specific Non-Meat Proteins and Peptides in Beef Burgers. *Molecules* **2018**, *24* (1), 18. https://doi.org/10.3390/molecules24010018.
(12) Fiorino, G. M.; Fresch, M.; Brümmer, I.; Losito, I.; Arlorio, M.; Brockmeyer, J.; Monaci, L. Mass Spectrometry-Based Untargeted Proteomics for the Assessment of Food Authenticity: The Case of Farmed Versus Wild-Type Salmon. *J. AOAC Int.* **2019**, *102* (5), 1339–1345. https://doi.org/10.5740/jaoacint.19-0062.
(13) Maia, T. M.; Staes, A.; Plasman, K.; Pauwels, J.; Boucher, K.; Argentini, A.; Martens, L.; Montoye, T.; Gevaert, K.; Impens, F. *A Simple Approach for Accurate Peptide*

Quantification in MS-Based Proteomics; preprint; Biochemistry, **2019**. https://doi.org/10.1101/703397.
(14) Majumdar, S.; Pagano, L.; Wohlschlegel, J. A.; Villani, M.; Zappettini, A.; White, J. C.; Keller, A. A. Proteomic, Gene and Metabolite Characterization Reveal the Uptake and Toxicity Mechanisms of Cadmium Sulfide Quantum Dots in Soybean Plants. *Environ. Sci. Nano* **2019**, *6* (10), 3010–3026. https://doi.org/10.1039/C9EN00599D.
